# Linear isomer but not branched isomers of perfluorooctane sulfonate in plasma is associated with eicosapentaenoic acid, a seafood consumption biomarker

**DOI:** 10.1265/ehpm.24-00140

**Published:** 2024-07-18

**Authors:** Zhaoqing Lyu, Kouji H. Harada, Mariko Harada Sassa, Yukiko Fujii

**Affiliations:** 1 Department of Health and Environmental Sciences, Kyoto University Graduate School of Medicine, Yoshida Konoe, Sakyo, Kyoto 606-8501, Japan; 2 Department of Pharmaceutical Sciences, Daiichi University of Pharmacy, 22-1 Tamagawa, Minami-ku, Fukuoka 815-8511, Japan

**Keywords:** Per- and polyfluoroalkyl substances, Structural isomer, Eicosapentaenoic acid-to-arachidonic acid ratio, Seafood consumption, Japanese

## Abstract

**Background:**

Per- and polyfluoroalkyl substances (PFAS) are global contaminants. Seafood consumption is a possible PFAS exposure route to humans while the isomer specific analysis has not been conducted.

**Methods:**

Perfluorooctane sulfonate (PFOS), perfluoroheptane sulfonate (PFHpS) and perfluorohexane sulfonate (PFHxS) were investigated in residents of Kyoto, Japan (n = 51). The relationship between plasma PFAS and seafood consumption biomarker, the ratio of eicosapentaenoic acid to arachidonic acid (EPA/AA) was examined by multiple regression analysis.

**Results:**

Linear PFOS concentrations showed a significant positive correlation with the EPA/AA ratio in plasma samples (β = 6.80, p = 0.0014). Linear PFHpS was marginally associated with EPA/AA ratio (β = 0.178, p = 0.0874). Branched PFOS isomers and PFHxS had no associations with EPA/AA ratios.

**Conclusion:**

Seafood intake may be a significant exposure pathway for PFAS, such as PFOS but the isomers differ.

**Supplementary information:**

The online version contains supplementary material available at https://doi.org/10.1265/ehpm.24-00140.

## Graphical Abstract

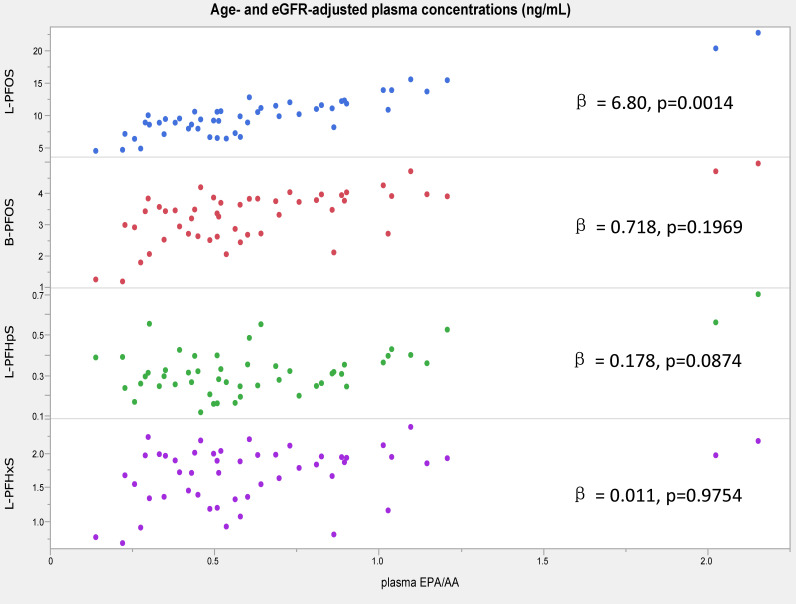

Per- and polyfluoroalkyl substances (PFAS) are highly stable fluorinated organic chemicals that have been utilized across various industries. Perfluorooctane sulfonate (PFOS) and perfluorooctanoic acid (PFOA) are known for their persistence and bioaccumulation in the environment and wildlives. Seafood can be contaminated with PFAS [1] and the relationship of the seafood consumption with blood PFAS concentration has been investigated in the Japanese population [2, 3]. Our previous research demonstrated that the plasma concentrations of long-chain perfluoroalkyl carboxylic acids (PFCAs, members of the PFAS family) with ≥8 carbon atoms, such as PFOA, were associated with the ratio of eicosapentaenoic acid (EPA, an omega-3 fatty acid) to arachidonic acid (AA, an omega-6 fatty acid), as a seafood consumption biomarker, in residents of Kyoto, Japan [3]. However, short-chain substitute of PFOS, perfluorohexane sulfonate (PFHxS) with 6 carbon atoms was not examined, which is restricted under the Stockholm Convention on Persistent Organic Pollutants in 2022 due to their potential impact on health. In addition, PFOS consists of 11 major structural isomers with different physicochemical properties. In the previous study by Yamaguchi et al. [2], isomers of PFOS were not distinguished. Therefore, in the present study, we evaluated the relationships between the plasma concentrations of these PFAS and the EPA/AA ratio in a Japanese population.

The analyzed plasma samples (n = 51) in the current study were subsampled randomly from the initial samples of the previous study (n = 131) [3]. Characteristics of the participants are shown in Table S1. Plasma concentrations of PFOS (C_8_F_17_SO_3_^−^), perfluoroheptane sulfonate (C_7_F_15_SO_3_^−^, PFHpS, impurities of PFOS or PFHxS), PFHxS (C_6_F_13_SO_3_^−^) and their isomers were measured by gas chromatography-chemical ionization-mass spectrometry as reported in a previous study [4]. The list of analyzed PFAS, their limits of detection (LODs) and detection frequencies are presented in Table S2. Participant recruitment, blood sample collection and chemical analysis procedures are summarized in the Supplementary Material. In the statistical analysis, compounds with detection frequencies less than 20% were excluded. Concentrations less than LODs were set as LOD/2. Concentrations of branched PFOS isomers were calculated as the sum of them. Multiple linear regression was performed to examine the associations between plasma PFAS levels and EPA/AA. Since renal clearance has been reported as a primary elimination pathway for PFAS, we incorporated age and estimated glomerular filtration rate (eGFR) as covariates in the analysis [3]. The analysis was conducted using JMP Pro Statistical Software, version 17 (SAS institute, Cary, NC, USA).

Distribution of plasma PFAS concentrations is summarized in Table S3. PFOS were detected in all plasma samples, composed with 80.2% linear and 19.8% branched PFOS by concentration. Linear PFHxS was detected in 50 samples with a detection frequency of 98%. While only the isomer 4m-PFHxS detected in one sample, branched PFHxS were excluded from the analysis due to low detection frequency. Linear PFHpS was detected at 62.7% and mean concentration was lower than PFOS and PFHxS. Results of multiple linear regression were shown in Table 1. Only the concentrations of linear PFOS showed a significantly positive correlation with EPA/AA ratio (β = 6.80, 95% CI [2.77, 10.8], p = 0.0014). The association was evident at age ≤60 years (Table S4). Linear PFHpS was marginally associated with EPA/AA ratio (β = 0.178, p = 0.0874). The concentrations of linear PFOS, branched PFOS and linear PFHxS also showed positive associations with age, and linear PFHpS with eGFR (p < 0.05).

**Table 1:** Associations between plasma PFAS concentrations and EPA/AA adjusted with age and eGFR

		**L-PFOS**	**B-PFOS**	**L-PFHpS**	**L-PFHxS**
EPA/AA	β	6.80	0.718	0.178	0.011
	95% CI	(2.77, 10.8)	(−0.385, 1.82)	(−0.0271, 0.383)	(−0.719, 0.741)
	p	0.0014	0.1969	0.0874	0.9754

Age	β	0.136	0.039	0.00497	0.029
	95% CI	(0.0001, 0.272)	(0.001, 0.076)	(−0.00193, 0.0119)	(0.004, 0.053)
	p	0.0498	0.0425	0.1541	0.0223

eGFR	β	0.042	−0.014	0.00723	−0.002
	95% CI	(−0.067, 0.151)	(−0.044, 0.016)	(0.00167, 0.0128)	(−0.022, 0.018)
	p	0.4423	0.3457	0.0119	0.8219

R^2^		0.317	0.261	0.169	0.171

Regression coefficients (β) are expressed as point estimate (95% confidence interval [CI]).

Possible explanation to the difference between isomers is reasoned by the biological half-lives of branched PFOS isomers, which would be shorter than that of linear PFOS [5]. The PFHxS level was not correlated with the EPA/AA ratio in the present study. Fish consumption was identified as a source of PFHxS exposure in one previous research [6] while other studies have reported no significant association with consumption of seafood [7]. PFHpS showed only a marginal association with EPA/AA. These findings suggest that carbon chain length (from C_6_ to C_8_) and alkyl chain structure (linear chain or branched chain) could be a determinant of PFAS accumulation in seafoods and exposure to humans. In conclusion, seafood consumption may be an exposure route for specific PFAS, such as PFOS but the isomers differ. This is probably due to different toxicokinetics in humans and wildlives.

## Abbreviations

PFAS: per- and polyfluoroalkyl substance
PFOS: perfluorooctane sulfonate
PFHpS: perfluoroheptane sulfonate
PFHxS: perfluorohexane sulfonate
EPA/AA: eicosapentaenoic acid-to-arachidonic acid ratio
eGFR: estimated glomerular filtration rate
LOD: limits of detection

## Supplementary information

**Additional file 1:** **Table S1.** Characteristics of the selected subgroup participants. **Table S2.** List of analytes and their limit of detection (LODs) and detection frequencies (n = 51). **Table S3.** Distribution of PFAS concentrations (ng/mL). **Table S4.** Associations between plasma PFOS concentrations and EPA/AA stratified by age over 60 years.

## Declarations

### Availability of data and material

The data are available from the corresponding author upon reasonable request.
