# Supplementary material for: Linear isomer but not branched isomers of perfluorooctane sulfonate in plasma is associated with eicosapentaenoic acid, a seafood consumption biomarker
Source: Environ Health Prev Med. 2024 Jul 18;29:35. doi: 10.1265/ehpm.24-00140 (PMC11273034; doi:10.1265/ehpm.24-00140)
Supplement: Supplementary file 1 — Additional file 1: Table S1. Characteristics of the selected subgroup participants. Table S2. List of analytes and their limit of detection (LODs) and detection frequencies (n = 51). Table S3. Distribution of PFAS concentrations (ng/mL). Table S4. Associations between plasma PFOS concentrations and EPA/AA stratified by age over 60 years. [file ehpm-29-035-s001.docx]

*Sample collection*

Blood samples were collected from residents living in Uji city, Kyoto Prefecture, Japan. A 1-day event was held as part of health promotion program at a community center in November 2013. Flyers were distributed to each household in the city (73,430 households and 187,577 persons), that described the timing of the event and the measurements that would be made. Five hundred citizens visited this program, and they were informed on this study in the main session that all the visitors attended. Inclusion criteria was adult without blood coagulation disorder, living in Kyoto prefecture. Persons with end-stage renal disease were excluded due to possible difference in toxicokinetics of PFASs. Finally, one hundred thirty-one people were recruited to the sampling. They provided their written informed consent prior to participation. Demographic data and clinical history were obtained through face-to-face interviews using a structured questionnaire. The analyzed plasma samples (n = 51) in the current study were randomly subsampled from the initial samples.

Blood samples of 10 mL were collected into two 5-mL vacuum collection tubes (Venoject EDTA-K; Terumo, Tokyo, Japan) from a median cubital vein by a physician or nurse. Plasma samples were obtained by centrifugation at 3,000 × *g* for 15 min and stored at −30°C at the Kyoto Human Specimen Bank until analyzed [1].

*Chemical analysis of PFAS and fatty acids, and creatinine*

The concentrations of PFOS, PFHxS and their isomers were measured. The analyte list is presented in Table S2.

Plasma samples were subjected to gas chromatography to determine PFAS concentrations (2). To 0.10 mL of serum sample, 0.4 mL of acetonitrile and surrogate standard solution (EPA-533ES, Wellington Laboratories) were added, centrifuged to remove protein and the supernatant was allowed to dry. Bis(4-tert- (4-tert-phenyl)iodonium hexafluorophosphate (tBPI, Tokyo Kasei Kogyo) was used as the reagent for in-port arylation. Each sample extract was dissolved in a 1% (w/v) tBPI/Acetone solution and used for injection to GC-MS. PFAS was separated in an Agilent 6890 GC/5973 MSD inert using DB-5MS (30 m x 0.25 mm, 0.25 μm thickness) with splitless injection at an inlet temperature of 300°C. PFAS were measured under chemical ionization (methane gas, negative ionization mode).

Concentrations of fatty acids and creatinine in plasma samples were determined previously (3). Eicosapentaenoic acid (EPA) and arachidonic acid (AA) were extracted and measured using the method described in the aforementioned publication, and separated using a 6890GC and HP-5MS (Agilent Technologies, Santa Clara, CA, USA) (3). An enzymatic assay using creatinine amidohydrolase was used to determine the plasma creatinine concentration. The Estimated glomerular filtration rate (eGFR) (mL/min/1.73 m^2^) was calculated using the age, sex, and serum creatinine concentrations of the participants (4).

Table S1. Characteristics of the selected subgroup participants.

| Characteristic | |  | N (%) or mean (SD), median |
| --- | --- | --- | --- |
| Total number | |  | 51 (100%) |
| Sex | Male | | 15 (29%) |
|  | Female | | 36 (71%) |
| Age (year) | |  | 62.4 (13.7), 67 |
| Height (cm) | |  | 158.5 (8.9), 157 |
| Weight (kg) | |  | 55.0 (9.0), 54.1 |
| Serum creatinine (mg/dl) | |  | 0.674 (0.151), 0.65 |
| eGFR (mL/min/1.73 m^2^) | |  | 77.9 (16.6), 75 |
| EPA/AA ratio | |  | 0.654 (0.394), 0.562 |
| Smoking | No | | 38 (74%) |
|  | Ex-smoker | | 8 (16%) |
|  | Yes | | 5 (10%) |
| Alcohol consumption | No | | 21 (41%) |
|  | Ex-drinker | | 5 (10%) |
|  | Yes | | 25 (49%) |

SD, standard deviation; EPA/AA, eicosapentaenoic acid-to-arachidonic acid ratio; eGFR, estimated glomerular filtration rate.

Table S2. List of analytes and their limit of detection (LODs) and detection frequencies (n = 51).

| Abbreviation | Analyte | LOD (ng/L) | Detection frequency |
| --- | --- | --- | --- |
| L-PFHxS | Perfluoro-n-hexane sulfonate | 0.05 | 98.0% |
| 4m-PFHxS | Perfluoro-4-methyl-pentane sulfonate | 0.01 | 2.0% |
| 3m-PFHxS | Perfluoro-3-methyl-pentane sulfonate | 0.01 | 0% |
| 2m-PFHxS | Perfluoro-2-methyl-pentane sulfonate | 0.01 | 0% |
| 1m-PFHxS | Perfluoro-1-methyl-pentane sulfonate | 0.01 | 0% |
| L-PFHpS | Perfluoro-n-heptane sulfonate | 0.01 | 62.7% |
| L-PFOS | Perfluoro-n-octane sulfonate | 0.15 | 100% |
| 1m-PFOS | Perfluoro-1-methyl-heptane sulfonate | 0.02 | 84.3% |
| 2m-PFOS | Perfluoro-2-methyl-heptane sulfonate | 0.04 | 29.4% |
| 3m-PFOS | Perfluoro-3-methyl-heptane sulfonate | 0.05 | 98.0% |
| 4m-PFOS | Perfluoro-4-methyl-heptane sulfonate | 0.07 | 100% |
| 5m-PFOS | Perfluoro-5-methyl-heptane sulfonate | 0.07 | 98.0% |
| 6m-PFOS | Perfluoro-6-methyl-heptane sulfonate | 0.1 | 98.0% |
| 3,5dm-PFOS | Perfluoro-3,5-dimethyl-hexane sulfonate | 0.01 | 23.5% |
| 4,5dm-PFOS | Perfluoro-4,5-dimethyl-hexane sulfonate | 0.01 | 0% |
|  |  |  |  |
| PFHpA | Perfluoroheptanoic acid | 0.01 | 98% |
| PFOA | Perfluorooctanoic acid | 0.01 | 100% |
| PFNA | Perfluorononanoic acid | 0.01 | 100% |
| PFDA | Perfluorodecanoic acid | 0.01 | 100% |
| PFUnDA | Perfluoroundecanoic acid | 0.02 | 100% |
| PFDoDA | Perfluorododecanoic acid | 0.02 | 100% |
| PFTrDA | Perfluorotridecanoic acid | 0.04 | 100% |

LOD, limit of detection.

Table S3. Distribution of PFAS concentrations (ng/mL).

|  | Linear PFOS | Branched PFOS | Linear PFHpS | Linear PFHxS |
| --- | --- | --- | --- | --- |
| Minimum | 2.42 | 0.594 | <0.01 | <0.05 |
| 25th percentile | 7.91 | 2.20 | <0.01 | 0.946 |
| Median | 12.6 | 3.16 | 0.34 | 1.63 |
| 75th percentile | 18.0 | 4.04 | 0.56 | 2.23 |
| Maximum | 35.8 | 9.04 | 0.92 | 4.89 |
| Mean (SD) | 13.4 (7.36) | 3.30 (1.62) | 0.325 (0.284) | 1.70 (1.02) |
| Detection frequency | 100% | 100% | 62.7% | 98% |

|  | 1m-PFOS | 2m-PFOS | 3m-PFOS | 4m-PFOS | 5m-PFOS | 6m-PFOS | 3,5dm-PFOS | 4,5dm-PFOS |
| --- | --- | --- | --- | --- | --- | --- | --- | --- |
| Minimum | <0.02 | <0.04 | <0.05 | 0.12 | <0.07 | <0.10 | <0.01 | <0.01 |
| 25th percentile | 0.29 | <0.05 | 0.20 | 0.29 | 0.57 | 0.54 | <0.01 | <0.01 |
| Median | 0.47 | 0.06 | 0.27 | 0.47 | 0.95 | 0.87 | <0.01 | <0.01 |
| 75th percentile | 0.63 | 0.09 | 0.39 | 0.66 | 1.25 | 1.13 | <0.01 | <0.01 |
| Maximum | 0.95 | 0.24 | 0.60 | 2.41 | 3.73 | 2.04 | 0.51 | <0.01 |
| Mean (SD) | 0.44 (0.24) | 0.07 (0.04) | 0.30 (0.14) | 0.53 (0.37) | 1.02 (0.64) | 0.87 (0.40) | 0.08 (0.15) | - |
| Detection frequency | 84.3% | 29.4% | 98.0% | 100% | 98.0% | 98.0% | 23.5% | 0% |

|  | PFHpA | PFOA | PFNA | PFDA | PFUnDA | PFDoDA | PFTrDA |
| --- | --- | --- | --- | --- | --- | --- | --- |
| Minimum | <0.01 | 0.429 | 0.638 | 0.151 | 0.242 | 0.041 | 0.086 |
| 25th percentile | 0.039 | 2.7 | 1.88 | 0.433 | 0.583 | 0.085 | 0.156 |
| Median | 0.052 | 4.06 | 2.52 | 0.641 | 0.871 | 0.115 | 0.183 |
| 75th percentile | 0.077 | 6.15 | 3.28 | 0.912 | 1.16 | 0.164 | 0.216 |
| Maximum | 0.201 | 10.2 | 9.21 | 4.12 | 2.34 | 0.354 | 0.693 |
| Mean (SD) | 0.061 (0.036) | 4.36 (2.22) | 2.91 (1.71) | 0.741 (0.584) | 0.951 (0.514) | 0.137 (0.073) | 0.204 (0.094) |
| Detection frequency | 98% | 100% | 100% | 100% | 100% | 100% | 100% |

SD, standard deviation.

Detection frequency of branched PFOS means that at least one branched isomer was detected.

**Table S4.** Associations between plasma PFOS concentrations and EPA/AA stratified by age over 60 years.

|  |  | L-PFOS  (Age: >60 year, n=33) | L-PFOS  (Age: ≤60 year, n=18) |
| --- | --- | --- | --- |
| EPA/AA | β | 6.26 | 13.3 |
|  | 95% CI | (1.30, 11.2) | (4.74, 21.8) |
|  | p | 0.0151 | 0.0049 |
|  |  |  |  |
| Age | β | 0.200 | 0.267 |
|  | 95% CI | (-0.184, 0.584) | (0.0649, 0.468) |
|  | p | 0.295 | 0.0133 |
|  |  |  |  |
| eGFR | β | -0.0475 | 0.122 |
|  | 95% CI | (-0.240, 0.145) | (0.00897,0.236) |
|  | p | 0.618 | 0.0363 |
|  |  |  |  |
| R^2^ |  | 0.248 | 0.585 |

Regression coefficients (β) are expressed as point estimate (95% confidence interval [CI]).

References:

1. Koizumi A, Harada KH, Inoue K, Hitomi T, Yang HR, Moon CS, et al. Past, present, and future of environmental specimen banks. Environ Health Prev Med. 2009;14(6):307–18. doi: 10.1007/s12199-009-0101-1.
2. Harada KH, Fujii Y, Zhu J, Zheng B, Cao Y, Hitomi T. Analysis of Perfluorooctanesulfonate Isomers and Other Perfluorinated Alkyl Acids in Serum by In-Port Arylation Gas Chromatography Negative Chemical Ionization-Mass Spectrometry. Environ. Sci. Technol. Lett. 2020;7:259-65.
3. Soleman SR, Li M, Fujitani T, Harada KH. Plasma eicosapentaenoic acid, a biomarker of fish consumption, is associated with perfluoroalkyl carboxylic acid exposure in residents of Kyoto, Japan: a cross-sectional study. Environ Health Prev Med. 2023;28:38. doi: 10.1265/ehpm.22-00302.
4. Matsuo S, Imai E, Horio M, Yasuda Y, Tomita K, Nitta K, et al. Revised equations for estimated GFR from serum creatinine in Japan. Am J Kidney Dis. 2009;53(6):982-92; doi:10.1053/j.ajkd.2008.12.034.
